# Effects of a *Lactobacillus salivarius* mixture on performance, intestinal health and serum lipids of broiler chickens

**DOI:** 10.1371/journal.pone.0175959

**Published:** 2017-05-01

**Authors:** Parisa Shokryazdan, Mohammad Faseleh Jahromi, Juan Boo Liang, Kalavathy Ramasamy, Chin Chin Sieo, Yin Wan Ho

**Affiliations:** 1 Institute of Tropical Agriculture, Universiti Putra Malaysia, UPM, Serdang, Selangor, Malaysia; 2 Faculty of Pharmacy, Universiti Teknologi MARA, Puncak Alam, Selangor, Malaysia; 3 Institute of Bioscience, Universiti Putra Malaysia, UPM, Serdang, Selangor, Malaysia; University of Palermo, ITALY

## Abstract

The ban or severe restriction on the use of antibiotics in poultry feeds to promote growth has led to considerable interest to find alternative approaches. Probiotics have been considered as such alternatives. In the present study, the effects of a *Lactobacillus* mixture composed from three previously isolated *Lactobacillus salivarius* strains (CI1, CI2 and CI3) from chicken intestines on performance, intestinal health status and serum lipids of broiler chickens has been evaluated. Supplementation of the mixture at a concentration of 0.5 or 1 g kg^-1^ of diet to broilers for 42 days improved body weight, body weight gain and FCR, reduced total cholesterol, LDL-cholesterol and triglycerides, increased populations of beneficial bacteria such as lactobacilli and bifidobacteria, decreased harmful bacteria such as *E*. *coli* and total aerobes, reduced harmful cecal bacterial enzymes such as β-glucosidase and β-glucuronidase, and improved intestinal histomorphology of broilers. Because of its remarkable efficacy on broiler chickens, the *L*. *salivarius* mixture could be considered as a good potential probiotic for chickens, and its benefits should be further evaluated on a commercial scale.

## Introduction

The use of probiotics as an alternative to antibiotic growth promoters has attracted considerable interest due to its beneficial impacts on the health, performance and productivity of chickens [1,2,3,4,5]. Probiotics or direct-fed microbials are ‘live microbial supplements which beneficially affect the health of the host animal by improving its intestinal microbial balance’ [6].

Lactic acid bacteria, particularly *Lactobacillus* strains, are frequently used as probiotics [7]. *Lactobacillus* strains have a high ability to attach to the intestinal epithelium and are able to establish in the chicken intestine within a day after hatching [8], so they are considered to be normal bacterial flora of the gastrointestinal tract (GIT) of chickens [9]. Bacterial strains used as probiotics for animals should be isolated from the natural GIT microflora of the same type of animal in order to have more specific application [9].

Although different probiotics may be developed for different purposes, a potential probiotic strain intended for chickens mostly is developed towards improving the performance, general health and productivity of chickens, which are usually achieved by affecting intestinal microbial populations, serum lipids and intestinal morphology [1,5,10,11,12]. It has been reported that probiotic strains can help to maintain the microbial balance in the GIT as well as make changes in the composition of the intestinal microflora by increasing beneficial bacteria and decreasing harmful pathogens [13]. This could be due to competitive exclusion by competing for nutrients and attachment sites on the intestinal epithelial wall, or production of antimicrobial substances by probiotic strains or a synergy of both actions [6,11,14,15].

In terms of cholesterol lowering effects of probiotics, several mechanisms have been proposed, which are based on reduction of cholesterol synthesis or increase in degradation and excretion of cholesterol [16]. It has been also reported that some probiotic strains with BSH activity are able to reduce serum cholesterol through deconjugation of bile salts [17]. In addition, some probiotic cultures have been reported to be able of improving the morphology of chicken intestine toward increasing nutrient absorption and endogenous digestive enzymes secretion surface [18,19].

On the other hand, in terms of safety aspects, potential probiotic strains must not produce harmful toxic enzymes such as β-glucosidase and β-glucuronidase, which can cause toxic compounds being released in the colon.

In an earlier study [20], we have isolated several *Lactobacillus* strains from the intestines of chickens, identified the strains and assessed (*in vitro*) their ability to survive and colonize the GIT. Three strains (*L*. *salivarius* CI1, CI2 and CI3), which exhibited good probiotic properties such as tolerance to acid, bile and pancreatic enzymes, and a strong ability to adhere to the intestinal epithelial cells were selected as potential probiotics for chickens [20]. In the present study, the *in vivo* effects of these three *L*. *salivarius* strains (as a mixture) on the growth performance, cecal microbial populations, serum lipids, organ weights, intestinal villus and crypt lengths and harmful cecal bacterial enzyme activities (β-glucosidase and β-glucuronidase) of broiler chickens were investigated to confirm their potential as an effective probiotic mixture for chickens.

## Materials and methods

### Preparation of *Lactobacillus* cultures

The three *L*. *salivarius* strains (CI1, CI2 and CI3) were cultured separately in MRS broth medium (Merck, Darmstadt, Germany) and incubated for 24 h at 37°C in anaerobic jars (Oxoid, Basingstoke, UK) containing gaspack (AnaeroGen, Oxoid, UK). After incubation, the cultures were centrifuged at 5000 × *g* for 10 min at 4°C. Supernatants were discarded and cell pellets were washed three times with deionized water. The cell pellet of each *L*. *salivarius* strain was freeze-dried separately, and then mixed together in the ratio of 1:1:1 (w:w:w at 1×10^9^ CFU g^-1^). The mixture of *Lactobacillus* cultures was stored at -20°C and used daily as a dietary supplement for broiler chickens.

### Chickens and diets

Two hundred and seventy one-d-old male broiler chicks (Cobb 500), obtained from a local commercial hatchery, were used in this experiment. The chicks were housed in stainless steel, three-tiered battery cages (0.9 m length, 0.6 m width and 0.6 m height) with raised wire netted floors in an open house under natural tropical conditions. From d 1 to 14, the wire netted floors of the cages were lined with papers, which were changed daily. After that, sliding stainless steel trays were placed under the cages to collect feces, which were removed daily. For the first 14 d, chicks were brooded with a 100 W bulb.

The chicks were weighed on per cage basis and randomly allocated to three dietary treatment groups. Each dietary treatment consisted of six replicate cages of 15 chicks per cage. The dietary treatments were: (i) basal diet (control) (ii) basal diet + 1 g kg^-1^ of mixture of three *L*. *salivarius* strains (LC) and (iii) basal diet + 0.5 g kg^-1^ LC. The basal diet was an antibiotic-free, corn-soybean meal diet (Table 1) formulated to meet the nutrient requirements for starter (1 to 21 d) and grower (22 to 42 d) periods [21]. The feed was in a mash form, and was fed to chickens twice daily at 09:00 h and 17:00 h, in the way that chickens had ad libitum access to the feed. The mixture of *L*. *salivarius* strains (LC) was mixed in the feed daily using a feed mixer machine. The viability of the *Lactobacillus* cells was checked biweekly using conventional spread plate method. The experimental period was 42 d. The study was approved by the Ethics Committee of the Universiti Putra Malaysia, and the care and management of chickens and sampling procedures were in compliance with the guidelines of the Federation of Animal Science Societies [22]. Besides, the animals’ health and welfare were monitored by a qualified poultry veterinarian who is a member of the research team.

**Table 1:** Composition of basal diet.

Ingredient (g kg^-1^ unless otherwise stated)	Starter (1 to 21 d)	Grower (22 to 42 d)
Ground yellow corn	538.9	603.0
Soyabean meal	361.9	318.6
Fish meal	30.0	30.0
Palm oil	37.4	24.5
60% choline chloride	2.5	2.0
Trimix*	1.0	1.0
Salt (NaCl)	2.0	1.0
DL-methionine	1.8	0.4
Limestone	13.0	13.0
Dicalcium phosphate	11.5	6.5
Total	1000.0	1000.0
Calculated analysis (g kg^-1^ except energy)		
Methionine	9.5	8.5
Lysine	13.7	12.0
Crude protein	220.0	199.9
Crude fat	63.1	52.2
Crude fibre	38.0	36.5
Calcium	10.2	9.0
Phosphorus	4.5	3.5
Metabolisable energy (MJ kg^-1^)	13.06	13.06

* Trimix (per kg Trimix): iron 100 g; manganese 110 g; copper 20 g; zinc 100 g; iodine 2 g; selenite 0.2 g; cobalt 0.6 g; santoquin 0.6 g; folic acid 0.33 g; thiamin 0.83 g; pyridoxine 1.33 g; biotin 2% 0.03 g; riboflavin 2 g; cyanocobalamin 0.03 g; D-calcium pantothenate 3.75 g; niacin 23.3 g; retinol 2000 mg; cholecalciferol 25 mg; α-tocopherol 23,000 mg IU.

### Sample collection and analysis

Feed residual was collected once a day before morning feeding and feed consumption on per-cage basis was recorded daily. Body weight was recorded weekly, and body weight gain was calculated based on that; feed conversion ratio (FCR) was calculated as feed intake per weight gain unit. Mortality was recorded as it occurred, and the dead birds were immediately removed from the cages. At d 21 and 42, 18 chickens per treatment (three chickens per replicate cage) were randomly selected, weighed and euthanized by severing the jugular vein. Blood was collected in non-heparinized blood collection tubes to obtain the serum. The carcasses were opened immediately, and organs such as the heart, liver, spleen, bursa, and pancreas were removed and weighed. Small intestine was collected for villus and crypt length measurements, and cecal contents were collected for analyses of cecal microbial populations and determination of harmful cecal bacterial enzyme (β-glucosidase and β-glucuronidase) activities.

### Microbiological analyses of cecal contents

Cecal contents were analyzed for microbial populations using a conventional method (spread plate method) and a molecular technique (real-time PCR assay). For the conventional method, the cecal contents were used immediately after collection, however, for the molecular technique the cecal contents were preserved in -20°C until the day of assessment.

#### Conventional spread plate method

For the conventional spread plate method, 1 g of cecal content was suspended in 9 ml phosphate buffer saline (PBS) (8 g NaCl, 0.2 g KCl, 1.44 g Na_2_HPO_4_, 0.24 g KH_2_PO_4_ in 1 l distilled water, pH 7.2) and vortexed for 1 min. Samples were serially diluted in sterile diluents (0.5 g kg^-1^ peptone water in distilled water) and 100 μl of 10^−4^ to 10^−6^ dilutions were streaked on appropriate selective media for enumeration of different groups of bacteria. de Man, Rogosa and Sharpe (MRS) agar medium was used for enumeration of lactobacilli, Bifidus Selective agar for bifidobacteria, Brain-Heart Infusion agar for total aerobes, Brilliant Green agar for *Salmonella* and Eosin Methylene Blue agar for *E*. *coli* (all media from Sigma, Saint Louis, USA, except MRS from Merck). After incubation in appropriate conditions for each group of bacteria (72 h at 37°C in anaerobic condition for lactobacilli and bifidobacteria, and 48 h at 39°C in aerobic condition for *Salmonella*, *E*. *coli* and total aerobes), colonies on the plates were counted and microbial population was expressed as log_10_ CFU g^-1^ cecal content.

#### Real-time PCR assay

For quantitative real-time PCR assay, total DNA was extracted from cecal samples using the QIAamp DNA Stool Mini Kit (Qiagen Inc., USA). Quantification carried out based on the standard curve method in real-time PCR. The standard curves were constructed using number of copies of the 16S rRNA gene plotted against quantification cycle (Cq) obtained from 10-fold serial dilutions of PCR products from pure culture of each bacterial group. In order to prepare the standard curves, DNA was extracted from the pure culture of each target bacteria (*Lactobacillus*, *Bifidobacterium* and *E*. *coli*) and conventional PCR was used to amplify bacterial DNA. PCR products of the target bacteria were run in 1 g kg^-1^ agarose gel and specific bands were purified using the MEGAquick-spin^™^ purification kit (iNtRON Biotechnology, Korea). Purity and concentration of 16S rRNA gene in each sample was measured using a Nanodrop ND-1000 spectrophotometer (Implen NanoPhotometer^™^, Germany). The number of copies of the 16S rRNA gene per ml of elution buffer was calculated using the following formula that is available online (http://web.uri.edu/gsc/dsdna-calculator/):

$$\text{Number of copies}=\frac{\text{Amount of DNA }\left({\mu \text{g ml}}^{-1}\right)\;\times \;6.022\;\times \;10^{23}}{\text{Length }\left(\text{bp}\right)\times \;10^{9}\;\times \;650}$$

Since the efficiency of amplification among primers and templates may be variable, the amplification efficiency (E) of each primer-template combination was determined based on the slope value of the linear regression of each standard curve calculated by the following equation:

$$E\;\text{(}\%\text{)}\;=\;[10{\;}^{\left(-1/slope\right)}\;-\;1]\;\times \;100$$

In this equation, E is 100% if a 10-fold dilution of DNA template results in a Cq difference of 3.32.

Real-time PCR was performed with a BioRad CFX96 Real-time PCR system (BioRad, USA) using optical grade plates. Primers used in the quantification of different bacterial populations are shown in Table 2. The real-time PCR reaction was performed on a total volume of 25 μl using the Maxima SYBR Green qPCR Master Mix (Fermentas, USA). Each reaction consisted of 12.5 μl of 2 × SYBR Green Master Mix, 1 μl of 10 μM forward primer, 1 μl of 10 μM reverse Primer, 2 μl of DNA samples and 8.5 μl of nuclease-free water. Each sample was assayed with triplicate reactions. No-template control was included in the real-time PCR amplification to rule out any cross-contamination. Real-time PCR cycling conditions comprised an initial denaturation at 94°C for 5 min, followed by 40 cycles of denaturation at 94°C for 20 s, primer annealing at 58, 60 and 50°C for 30 s for *Lactobacillus*, *Bifidobacterium* and *E*. *coli*, respectively, and extension at 72°C for 20 s. Upon completion of the amplification, the specificity of the amplified product was confirmed by melting curve analysis. The real-time PCR products were incubated by raising the temperature from 70 to 95°C in 0.5°C increments with a hold of 5 s at each increment. The results were expressed as log_10_ copy number g^-1^ cecal content.

**Table 2:** Primers used for real-time PCR assay to target *Lactobacillus*, *Bifidobacterium* and *E*. *coli*.

Target group		Sequence 5′—3′	Reference
*Lactobacillus*	Forward	CATCCAGTGCAAACCTAAGAG	[23]
	Reverse	GATCCGCTTGCCTTCGCA	
*Bifidobacterium*	Forward	GGG TGG TAA TGC CGG ATG	[24]
	Reverse	TAA GCC ATG GAC TTT CAC ACC	
***E*. *coli***	Forward	GTGTGATATCTACCCGCTTCGC	[25]
	Reverse	AGAACGCTTTGTGGTTAATCAGGA	

### Serum lipid assay and relative weights of organs

Blood samples were allowed to settle at room temperature for 1 h, then centrifuged at 3000 × *g* for 10 min. The serum was transferred into vials and stored at -20°C until use. Serum samples were analyzed for total cholesterol, high density lipoprotein (HDL)-cholesterol, low density lipoprotein (LDL)-cholesterol and triglycerides using an automatic clinical chemistry analyzer (Hitachi, Japan).

The relative weight of organ was calculated as follows:

$$\text{Relative weight of organ }\left(\text{\%}\right)=\frac{\text{Weight of organ}}{\text{Live body weight}}\;\times 100$$

### Villus height and crypt depth measurements

A 1-cm segment of the midpoint of the jejunum was cut, gently washed with PBS and fixed in 100 ml l^-1^ formalin. Samples were then dehydrated for 16 h in an automatic tissue processor (Leica ASP 3000, Japan) and embedded in paraffin wax using a paraffin embedding system (Leica EG 1160, Japan). Each sample was cut into 4 μm-thick sections using a rotary microtome (Leica RM 2155, Japan). The sections were placed on glass slides, heated at 57°C until dried, then stained with haematoxylin and eosin. The stained sections were examined using a light microscope (Dialux, Leitz Wetzlar, Germany) fitted with a digital camera (Laica, Germany). Villus height was measured from the tip of the villus to the villus-crypt junction, while crypt depth was measured as the distance between the basement membrane and the mouth of crypt [26]. Fifteen measurements for villi and crypts were made for each sample.

### β-Glucosidase and β-glucuronidase activity assays

One gram of cecal contents was suspended in 10 ml of PBS (pH 7.2) and centrifuged at 3000 × *g* for 5 min. The supernatant was used for analysis of harmful cecal bacterial enzyme (β-glucosidase and β-glucuronidase) activities.

The assays for β-glucosidase and β-glucuronidase activities were according to that described by Lee *et al*. [27] with modifications. Briefly, 0.8 ml of 2 mM p-nitrophenyl-β-D-glucopyranoside (Sigma) (for β-glucosidase activity) or 2 mM p-nitrophenyl-β-D-glucuronide (Sigma) (for β-glucuronidase activity) and 0.2 ml of sample were incubated at 37°C for 1 h. The reaction was stopped by adding 1 ml of 0.5 mol l^-1^ NaOH, and the mixture was centrifuged at 4000 × g for 10 min at room temperature. Enzyme activity of the supernatant was determined by measuring absorbance at 405 nm using a spectrophotometer. Different concentrations (0, 0.1, 0.2, 0.5, 1 and 10 mmol l^-1^) of p-nitrophenol (Sigma) were used for preparation of a standard curve. The enzyme activity was expressed as unit g^-1^ cecal contents. One unit is defined as the activity required to release 1 μmol l^-1^ of p-nitrophenol in 1 h.

### Statistical analysis

All the data were analyzed using one-way ANOVA procedure of SAS program (2008) version 9.2. [28] based on the completely randomized design, followed by comparison among means using Duncan’s new multiple range test. Differences were considered significant if P < 0.05.

## Results

### Performance of broiler chickens

The effects of a mixture of *L*. *salivarius* CI1, CI2 and CI3 (LC) on body weight, body weight gain, feed intake and FCR of broiler chickens are shown in Table 3. The body weights of broiler chickens were not significantly different among the three dietary treatments at 1 and 21 d of age. However, at 42 d of age, chickens fed 0.5 or 1 g kg^-1^ LC showed significantly (P < 0.01) higher body weights (2164.3 and 2274.5 g, respectively) than control chickens (2017.3 g). From 1 to 21 d of age, body weight gains of broiler chickens were not significantly different among the dietary treatments, but from 22 to 42 and 1 to 42 d of age, broilers given 0.5 or 1 g kg^-1^ LC had significantly (P < 0.01) higher body weight gains than control chickens. There was no significant difference in feed intake of broilers in the three dietary treatments throughout the experimental period. From 1 to 21 d of age, the FCRs of all broilers were not significantly different. However, from 22 to 42 and 1 to 42 d of age, broiler chickens fed 0.5 or 1 g kg^-1^ LC had significantly (P < 0.01) better FCR than control chickens. Mortality was observed in the control and broilers supplemented with 0.5 g kg^-1^ LC (one chicken for each treatment group during 42 days of experiment), but there was no mortality in broilers fed 1 g kg^-1^ LC. During the experimental period, no significant difference (P > 0.05) was observed between the two groups of broiler receiving LC (0.5 or 1 g kg^-1^ LC) in terms of body weight, weight gain, feed intake or FCR.

**Table 3:** Effects of dietary treatments on body weight, body weight gain, feed intake and FCR of broiler chickens.

Parameter	Dietary treatment*		
	Control	0.5 g kg^-1^ LC	1 g kg^-1^ LC
Body weight (g)			
D 1	47.5 ± 1.0	47.2 ± 1.2	48.0 ± 1.4
D 21	644.7 ± 8.6	646.2 ± 18.7	653.8 ± 10.7
D 42	2017.3 ± 128.1 ^b^	2164.3 ± 172.3 ^a^	2274.5 ± 33.3 ^a^
Weight gain (g)			
1 to 21 d	597.2 ± 8.8	599.0 ± 18.1	605.8 ± 11.6
22 to 42 d	1348.3 ± 119.6 ^b^	1522.4 ± 192.0 ^a^	1615.8 ± 31.6 ^a^
1 to 42 d	1945.5 ± 119.1 ^b^	2121.4 ± 183.9 ^a^	2221.6 ± 26.3 ^a^
Feed intake (g)			
1 to 21 d	1020.2 ± 34.7	992.9 ± 36.9	992.1 ± 31.5
22 to 42 d	2798.7 ± 149.2	2661.2 ± 148.9	2713.5 ± 111.3
1 to 42 d	3818.9 ± 174.8	3654.1 ± 160.1	3705.6 ± 138.5
FCR (g g^-1^)			
1 to 21 d	1.71 ± 0.06	1.66 ± 0.04	1.64 ± 0.07
22 to 42 d	2.08 ± 0.15 ^a^	1.75 ± 0.17 ^b^	1.68 ± 0.05 ^b^
1 to 42 d	1.96 ± 0.10 ^a^	1.72 ± 0.11 ^b^	1.67 ± 0.05 ^b^

* Values are mean ± SD of 6 replicate cages, each with 15 chickens

^a-b^ Means within a row with no common superscript are significantly different (P < 0.01)

LC, mixture of *L*. *salivarius* CI1, CI2 and CI3 in the ratio of 1:1:1 (w:w:w); FCR, feed conversion ratio; control, basal diet; 0.5 g kg^-1^ LC, basal diet + 0.5 g kg^-1^ LC; 1 g kg^-1^ LC, basal diet + 1 g kg^-1^ LC

### Enumeration of cecal bacteria

#### Conventional microbiological method

**Fig 1:**
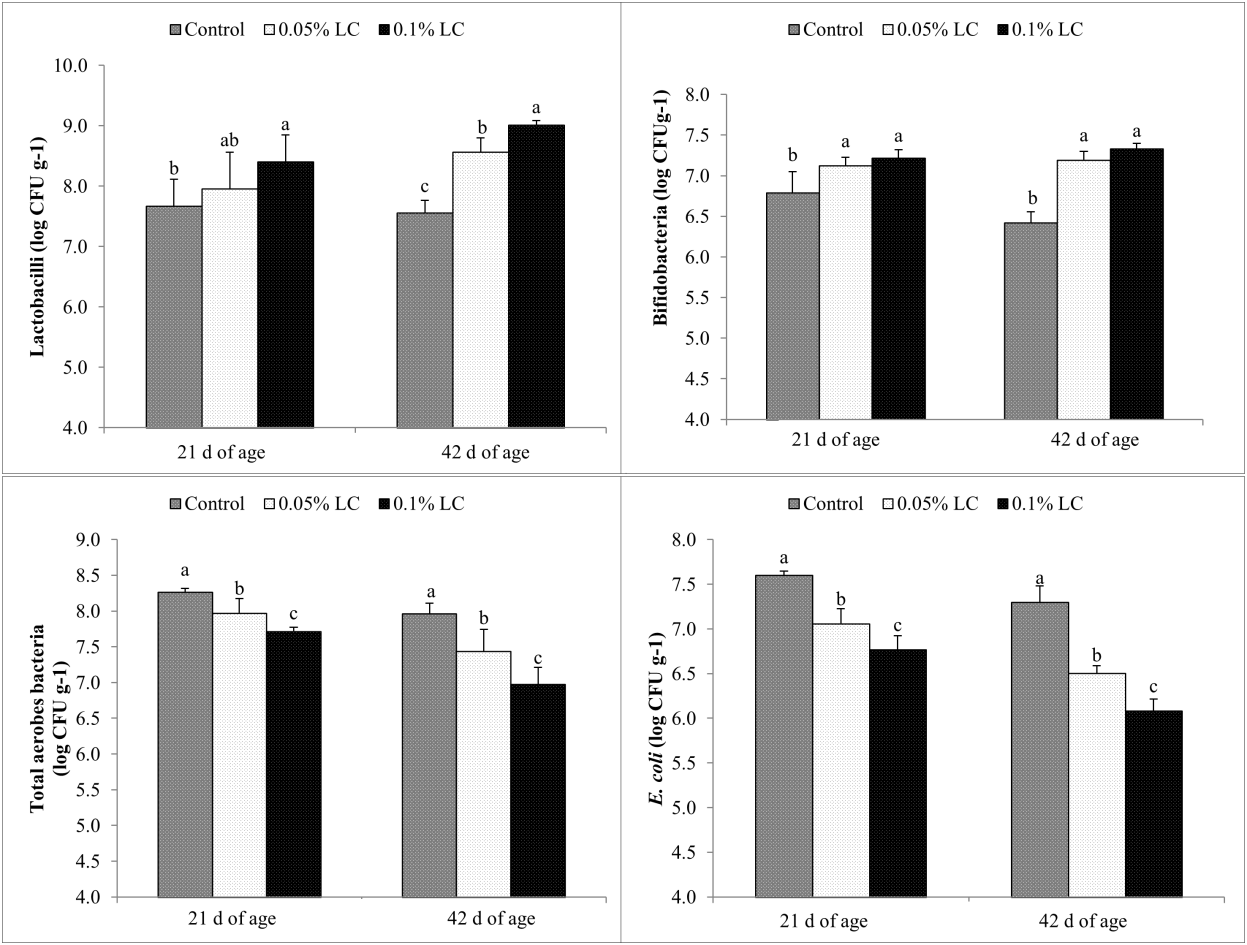
Effects of dietary supplementations of a mixture of three *L*. *salivarius* strains (LC) on populations of cecal lactobacilli, bifidobacteria, total aerobes and *E*. *coli* of broiler chickens at 21 and 42 d of age enumerated using the conventional spread plate method and expressed as log_10_ CFU g^-1^. Columns represent means of six birds in each treatment group (one chicken per replicate cage) ± SD. Within each period, columns with different letters differ significantly (P < 0.05). Control, basal diet; 0.05% LC, basal diet + 0.5 g kg^-1^ LC; 0.1% LC, basal diet + 1 g kg^-1^ LC.

Fig 1 shows the results of bacterial enumeration using the conventional spread plate method for lactobacilli, bifidobacteria, total aerobes and *E*. *coli*. No *Salmonella* was detected in the cecal contents of broiler chickens in all three dietary treatment groups throughout the experimental period. At 21 d of age, the population of lactobacilli in cecal contents of broiler chickens fed 1 g kg^-1^ LC was significantly (P < 0.05) higher than that of control chickens. Although the population of lactobacilli of chickens fed 0.5 g kg^-1^ LC was not significantly different (P > 0.05) from that of control chickens, it was numerically higher. At 42 d of age, the populations of lactobacilli in broiler chickens given 0.5 or 1 g kg^-1^ LC were significantly (P < 0.05) higher than that of the control, and between the two LC-supplemented groups, chickens given 1 g kg^-1^ LC showed significantly (P < 0.05) higher lactobacilli population than those fed 0.5 g kg^-1^ LC. At 21 and 42 d of age, broiler chickens supplemented with 0.5 or 1 g kg^-1^ LC had significantly (P < 0.05) higher populations of bifidobacteria than the control. At both ages, the cecal bifidobacterial populations between broilers fed 0.5 or 1 g kg^-1^ LC were not significantly different. Birds fed dietary treatments supplemented with 0.5 or 1 g kg^-1^ LC had significantly (P < 0.01) lower populations of total cecal aerobes than the control at 21and 42 d of age.

Between the two supplemented groups, birds fed 0.5 g kg^-1^ LC had significantly (P < 0.01) higher population of total cecal aerobes than those fed 1 g kg^-1^ LC at both ages. Broiler chickens supplemented with 0.5 or 1 g kg^-1^ LC had significantly (P < 0.01) lower populations of *E*. *coli* than control broilers at 21 and 42 d of age. At both ages, the *E*. *coli* populations of broilers given 1 g kg^-1^ LC were significantly (P < 0.01) lower when compared to those fed 0.5 g kg^-1^ LC.

#### Real-time PCR quantification

Since no *Salmonella* was detected in the cecal contents of broiler chickens in all three dietary treatment groups at 21 and 42 d of age using the conventional microbiological method, real-time PCR assay was not carried out for quantification of *Salmonella*. Real-time PCR quantification was also not conducted for total aerobes due to no existing designed primer for them. The standard curves for *Lactobacillus*, *Bifidobacterium* and *E*. *coli* were constructed using the plot of copy numbers of 16S rRNA gene of each bacterial group against its Cq values. The standard curves had high correlation coefficients of R^2^ = 0.988, 0.986 and 0.994 for *Lactobacillus*, *Bifidobacterium* and *E*. *coli*, respectively, indicating that the Cq values were proportional to the copy numbers of 16S rRNA gene, for each target bacterial group. From the slopes of the liner regressions of -3.407, -3.488 and -3.301, amplification efficiencies were obtained 96.6, 93.5 and 100.9% for *Lactobacillus*, *Bifidobacterium* and *E*. *coli*, respectively. The amplification curves for *Lactobacillus*, *Bifidobacterium* and *E*. *coli* were constructed by plotting the cycle numbers against fluorescence signals (RFU, relative fluorescence units). No fluorescence signals were detected from the no-template control. The melting temperatures of 82.5, 86 and 79.5°C were detected at which the sets of primers were specific for estimation of lactobacilli, bifidobacteria and *E*. *coli*, respectively.

**Fig 2:**
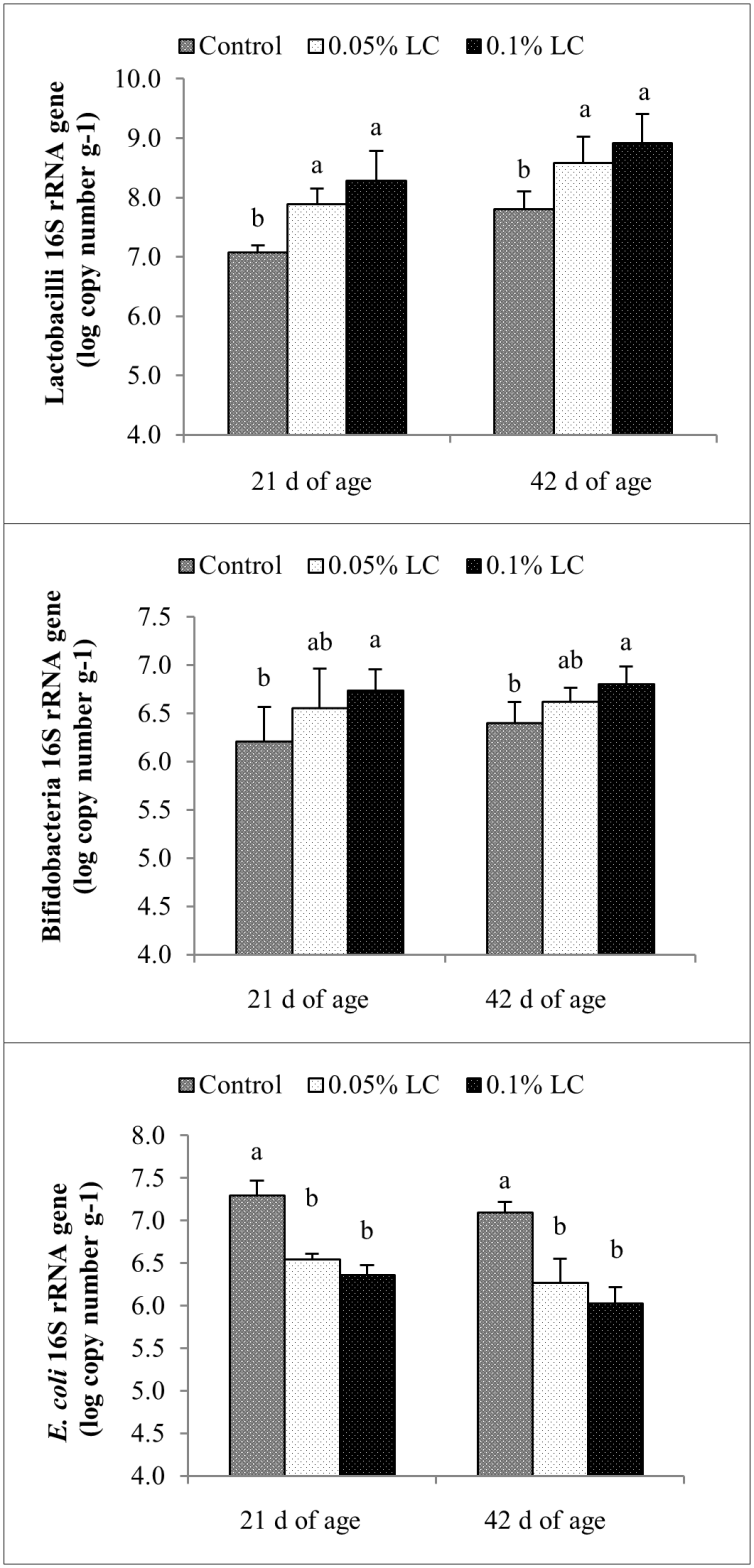
Effects of dietary supplementations of a mixture of *L*. *salivarius* strains (LC) on populations of cecal lactobacilli, bifidobacteria and *E*. *coli* of broiler chickens at 21 and 42 d of age quantified using real-time PCR and expressed as log_10_ copy number g^-1^. Columns represent means of six birds in each treatment group (one chicken per replicate cage) ± SD. Within each period, columns with different letters differ significantly (P < 0.05). Control, basal diet; 0.05% LC, basal diet + 0.5 g kg^-1^ LC; 0.1% LC, basal diet + 1 g kg^-1^ LC.

The results from real-time PCR quantification of cecal lactobacilli, bifidobacteria and *E*. *coli* populations of broiler chickens fed the three dietary treatments are shown in Fig 2. Cecal lactobacilli populations were significantly (P < 0.05) higher in broilers fed diets containing 0.5 or 1 g kg^-1^ LC when compared to that in control broilers at 21 and 42 d of age, and there was no significant difference between the cecal lactobacilli populations of broilers fed 0.5 or 1 g kg^-1^ LC.

At 21 and 42 d of age, broiler chickens fed 1 g kg^-1^ LC showed significantly (P < 0.05) higher cecal bifidobacteria populations than the control. However, at both ages, the cecal bifidobacteria populations of broilers supplemented with 0.5 g kg^-1^ LC were not significantly different from that of control or broilers fed 1 g kg^-1^ LC. Broiler chickens supplemented with 0.5 or 1 g kg^-1^ LC had significantly (P < 0.05) lower populations of *E*. *coli* than those of control broilers at 21 and 42 d of age, and the cecal *E*. *coli* populations between broilers fed 0.5 or 1 g kg^-1^ LC were not significantly different at both ages.

### Serum lipids and relative weights of organs

The results of serum lipid analysis of broilers fed the three dietary treatments at 21 and 42 d of age are shown in Table 4. Serum total cholesterol, LDL-cholesterol and triglyceride concentrations were significantly (P < 0.05) reduced in broiler chickens fed 0.5 or 1 g kg^-1^ LC when compared to control broilers at 21 and 42 d of age, and there was no significant difference between the two supplemented (0.5 or 1 g kg^-1^ LC) treatment groups at both ages. HDL-cholesterol levels of broilers were not significantly different in all three dietary treatment groups at 21 and 42 d of age.

**Table 4:** Effects of dietary supplementations of a mixture of *L*. *salivarius* strains on serum lipid concentrations of broiler chickens at 21 and 42 d of age.

Serum lipids	Dietary treatment*		
	Control	0.5 g kg^-1^ LC	1 g kg^-1^ LC
**21 d of age**			
**Total cholesterol (mg dL**^**-1**^**)**	133.80±14.41^a^	116.11±6.64^b^	114.61±6.49^b^
**HDL (mg dL**^**-1**^**)**	70.87±7.58	69.69±5.96	70.85±11.02
**LDL (mg dL**^**-1**^**)**	56.33±9.37^a^	45.83±2.86^b^	43.34±6.96^b^
**Triglycerides (mg dL**^**-1**^**)**	43.16±5.94^a^	37.28±2.95^b^	38.00±5.77^b^
**42 d of age**			
**Total cholesterol (mg dL**^**-1**^**)**	133.58±13.77^a^	109.30±1.08^b^	107.06±16.80^b^
**HDL (mg dL**^**-1**^**)**	66.78±7.15	68.05±12.94	70.62±13.11
**LDL (mg dL**^**-1**^**)**	56.97±6.05^a^	41.79±9.27^b^	42.97±7.86^b^
**Triglycerides (mg dL**^**-1**^**)**	52.64±8.84^a^	40.49±5.64^b^	39.73±7.85^b^

* Values are means ± SD of 6 replicate cages of 3 chickens each

^a—b^ Means within a row with no common superscript are significantly (P < 0.05) different

LC, mixture of *L*. *salivarius* CI1, CI2 and CI3 in the ratio of 1:1:1 (w:w:w); control, basal diet; 0.5 g kg^-1^ LC, basal diet + 0.5 g kg^-1^ LC; 1 g kg^-1^ LC, basal diet + 1 g kg^-1^ LC

The relative weights of organs calculated as percentage of body weight of broilers are given in Table 5. There were no significant differences in the relative weights of heart, liver, spleen, bursa and pancreas of broiler chickens from the three dietary treatment groups at 21 and 42 d of age.

**Table 5:** Effects of dietary supplementations of a mixture of *L*. *salivarius* strains on relative weights of organs of broiler chickens at 21 and 42 d of age.

Relative organ weight (%)	Dietary treatment*		
	Control	0.5 g kg^-1^ LC	1 g kg^-1^ LC
**21 d of age**			
**Heart**	0.58 ± 0.11	0.55 ± 0.04	0.56 ± 0.08
**Liver**	2.41 ± 0.24	2.38 ± 0.13	2.32 ± 0.25
**Spleen**	0.11 ± 0.02	0.10 ± 0.02	0.10 ± 0.02
**Pancreas**	0.35 ± 0.07	0.38 ± 0.05	0.35 ± 0.06
**Bursa**	0.21 ± 0.03	0.22 ± 0.04	0.20 ± 0.04
**42 d of age**			
**Heart**	0.44 ± 0.08	0.43 ± 0.05	0.44 ± 0.06
**Liver**	2.14 ± 0.15	2.01 ± 0.25	2.11 ± 0.28
**Spleen**	0.16 ± 0.03	0.16 ± 0.02	0.15 ± 0.03
**Pancreas**	0.19 ± 0.04	0.19 ± 0.03	0.19 ± 0.02
**Bursa**	0.18 ± 0.02	0.17 ± 0.01	0.16 ± 0.02

* Values are means ± SD of 6 replicate cages of 3 chickens each

LC, mixture of *L*. *salivarius* CI1, CI2 and CI3 in the ratio of 1:1:1 (w:w:w); control, basal diet; 0.5 g kg^-1^ LC, basal diet + 0.5 g kg^-1^ LC; 1 g kg^-1^ LC, basal diet + 1 g kg^-1^ LC

### Intestinal villus height and crypt depth

**Fig 3:**
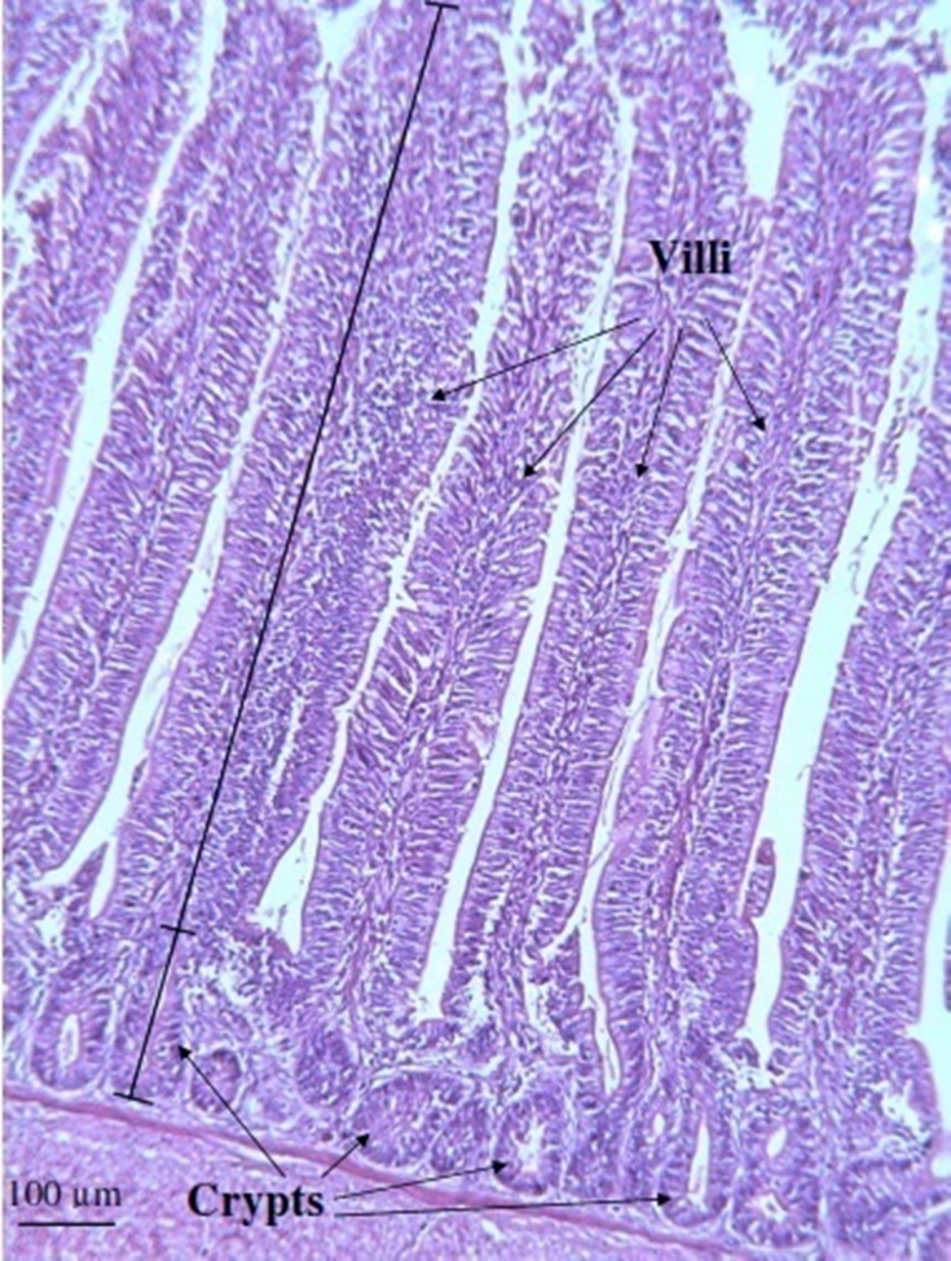
A representative photomicrograph showing intestinal villi and crypts of a broiler (fed diet supplemented with 1 g kg^-1^ of a mixture of *L*. *salivarius* CI1, CI2 and CI3) at 42 d of age. Villus height was measured from the top of the villus to the villus-crypt junction (long bar). Crypt depth was measured as the distance between the basement membrane and the mouth of crypt (short bar).

The effects of dietary treatments on intestinal villus height, crypt depth, and villus height:crypt depth ratio are presented in Table 6. At 21 d of age, there was no significant difference (P > 0.05) between the three dietary treatment groups in terms of villus heights and crypt depths. In broilers fed 1 g kg^-1^ LC, the villus height:crypt depth ratio was significantly (P < 0.05) higher than that of the control group (5.72 vs 5.15). However, birds fed 0.5 g kg^-1^ LC (5.54) did not showed any significant difference with the control birds for the villus height:crypt depth ratio. At 42 d of age, broilers given 0.5 or 1 g kg^-1^ LC showed significantly (P < 0.05) higher villus heights (1290.58 and 1312.50 μm, respectively) and villus height:crypt depth ratios (6.52 and 6.76, respectively) than control broilers (1110.17 μm villus height and 5.41 villus height:crypt depth ratio), and there was no significant difference between those fed 0.5 or 1 g kg^-1^ LC. However, crypt depths were not significantly different in broilers from the three dietary treatment groups. A representative photomicrograph of the intestinal villi and crypts of broilers showing measurements of villus height and crypt depth is given in Fig 3.

**Table 6:** Effects of dietary supplementations of a mixture of *L*. *salivarius* strains on intestinal villus height and crypt depth of broiler chickens at 21 and 42 d of age.

Parameters	Dietary treatment*		
	Control	0.5 g kg^-1^ LC	1 g kg^-1^ LC
**21 d of age**			
**Villus height (μm)**	1018.83 ± 65.60	1056.67 ± 44.40	1049.83 ± 75.82
**Crypt depth (μm)**	198.75 ± 19.12	191.83 ± 14.56	184.50 ± 21.63
**Villus: crypt ratio**	5.15 ± 0.42 ^b^	5.54 ± 0.49 ^a^^b^	5.72 ± 0.52 ^a^
**42 d of age**			
**Villus height (μm)**	1110.17 ± 82.02 ^b^	1290.58 ± 50.83 ^a^	1312.50 ± 91.13 ^a^
**Crypt depth (μm)**	206.50 ± 31.04	198.83 ± 17.35	195.17 ± 15.91
**Villus: crypt ratio**	5.41 ± 1.04 ^b^	6.52 ± 0.65 ^a^	6.76 ± 0.67 ^a^

* Values are means ± SD of 6 replicate cages of 3 chickens each

^a—b^ Means within a row with no common superscript are significantly (P < 0.05) different

LC, mixture of *L*. *salivarius* CI1, CI2 and CI3 in the ratio of 1:1:1 (w:w:w); control, basal diet; 0.5 g kg^-1^ LC, basal diet + 0.5 g kg^-1^ LC; 1 g kg^-1^ LC, basal diet + 1 g kg^-1^ LC

#### Harmful cecal bacterial enzyme (β-glucosidase and β-glucuronidase) activities

The cecal bacterial β-glucosidase and β-glucuronidase activities of broiler chickens fed the three dietary treatments are shown in Table 7. Supplementation of 0.5 or 1 g kg^-1^ LC to broiler chickens significantly (P < 0.01) decreased cecal β-glucosidase and β-glucuronidase activities at 21 and 42 d of age, and there was no significant difference in the bacterial enzyme activities between broilers fed 0.5 or 1 g kg^-1^ LC.

**Table 7:** Effects of dietary supplementations of a mixture of *L*. *salivarius* strains on β-glucuronidase and β-glucosidase activities in the cecal contents of broilers at 21 and 42 d of age.

Enzyme activity	Dietary treatment*		
	Control	0.5 g kg^-1^ LC	1 g kg^-1^ LC
**21 d of age**			
**β-Glucosidase activity (unit g**^**-1**^**)**	8.90± 1.61 ^a^	4.88± 1.21 ^b^	4.30 ± 0.83 ^b^
**β-Glucuronidase activity (unit g**^**-1**^**)**	1.66 ± 0.18 ^a^	1.26 ± 0.24 ^b^	1.14± 0.24 ^b^
**42 d of age**			
**β-Glucosidase activity (unit g**^**-1**^**)**	9.69 ± 0.89 ^a^	6.34± 0.71 ^b^	5.87± 0.86 ^b^
**β-Glucuronidase activity (unit g**^**-1**^**)**	3.47 ± 0.36 ^a^	2.17 ± 0.45 ^b^	2.01 ± 0.27 ^b^

* Values are mean ± SD of 6 replicate cages of 3 chickens each

^a–b^ Means within a row with no common superscript are significantly (P < 0.01) different

Unit, the activity required to release 1 μM of p-nitrophenol in 1 h; LC, mixture of *L*. *salivarius* CI1, CI2 and CI3 in the ratio of 1:1:1 (w:w:w); control, basal diet; 0.5 g kg^-1^ LC, basal diet + 0.5 g kg^-1^ LC; 1 g kg^-1^ LC, basal diet + 1 g kg^-1^ LC

## Discussion

Although improving effects of the mixture of three *L*. *salivarius* strains (CI1, CI2 and CI3) at 0.5 or 1 g kg^-1^ on performance of broilers from 1 to 21 d of age were not significant, supplementation of the mixture significantly improved body weight of broilers at 42 d of age; it also improved body weight gain and FCR of broilers from 22 to 42 and 1 to 42 d of age. A number of studies had also shown improvements in body weight and FCR of broiler chickens fed diets supplemented with a mixture of *Lactobacillus* strains [3,10,11,29,30,31,32] or with preparations of lactobacilli and other bacteria [5,33]. However, there were also some studies which reported no positive results in performance of broilers fed probiotic *Lactobacillus* supplemented feeds [34,35,36]. The variations in the results from different studies could be due to differences in the strains, sources, viability and concentrations of used bacteria, methods of administration, and conditions of chickens.

Supplementation of the three *L*. *salivarius* strains had no effect on feed intake of broiler chickens. Several other studies had also shown that feed intake of chickens was not affected by supplementation of *Lactobacillus* or other bacteria [3,5,10,11,29,30,31,32,37]. At present, it is not known why supplementation of *Lactobacillus* cultures to broiler chickens does not affect their feed intake. In layers, it has been reported that supplementation of *Lactobacillus* cultures stimulated their appetite [38,39,40]. However, this difference between broilers and layers may be attributed to the fact that broilers have been genetically selected for having high feed intake in comparison to layers, and as it has been reported by Ferket and Gernat [41], dietary factors are less important than management and flock health issues for influencing feed intake in broilers. Therefore, in unstressed broilers usually it is difficult to see the effects of dietary supplements on feed intake.

In the present study, two methods, namely, the conventional microbiological method (spread plate method) and the molecular technique (real-time PCR assay) were used to estimate cecal microbial populations of broilers fed the three dietary treatments. One of the weaknesses of the conventional microbiological method that has often been mentioned is that it may underestimate microbial populations as some of the microbes may be clumped together or lyzed during processing of samples. As real-time PCR assay measures microbial DNA, it may be a better approach for estimation of microbial populations. However, the results showed that population patterns of lactobacilli, bifidobacteria and *E*. *coli* obtained by the conventional microbial method and the real-time PCR assay were comparable.

Both enumeration methods showed that the *L*. *salivarius* strains had beneficial modulatory effects on the intestinal microflora of broilers fed 0.5 or 1 g kg^-1^ *Lactobacillus* strains, meaning that the populations of cecal beneficial bacteria (lactobacilli and bifidobacteria) were significantly increased, while populations of harmful bacteria (*E*.*coli* and total aerobes) were decreased. Jin *et al*. [42] and Saminathan *et al*. [3] reported similar beneficial modulation of intestinal microbial population in which there was an increase in intestinal lactobacilli and a decrease in *E*. *coli* of broilers fed a mixture of 12 *Lactobacillus* cultures at 21 d of age. Ngoc Lan *et al*. [43] also reported that two probiotic strains, *L*. *agilis* and *L*. *salivarius*, isolated from chicken intestine, significantly increased the intestinal lactobacilli in the probiotic group in comparison to the control group, after seven days of probiotic feeding. Gunal *et al*. [44] reported that a probiotic mixture (Protexin) decreased the population of Gram-negative bacteria in the ileal and cecal contents of broilers at 21 and 42 d of age. Mountzouris *et al*. [45] also used a probiotic mixture consisting of *L*. *reuteri*, *Enterococcus faecium*, *Bifidobacterium animalis*, *Pediococcus acidilactici* and *L*. *salivarius* and found that the populations of bifidobacteria, lactobacilli and gram-positive cocci were significantly higher in cecal contents of birds received probiotic (1 g kg^-1^ of feed) compared with the control chickens (receiving no additive in their feed) and chickens receiving antibiotic (avilamycin, 2.5 mg kg^-1^ of feed).

Although the precise mechanisms underlying the beneficial effects of probiotics are unclear, one of the proposed modes of action of probiotics is their pathogen interference and antagonistic activity, whereby probiotic strains inhibit the growth and colonization of other microorganisms, such as pathogens [7]. This could be due to competitive exclusion by competing for nutrients and attachment sites on the intestinal epithelial wall, or production of antimicrobial substances by probiotic strains or a synergy of both actions [6,11]. As a result, probiotic strains can help to maintain the gut health by providing a beneficial microbial balance in the GIT, and a healthy, well functioning gut with reduced digestive disorders would ensure better utilization and conversion of feeds, resulting in improved growth and vitality of the animal [13].

The results of the current study showed that both concentrations of the LC caused significant reduction in the serum total cholesterol and triglyceride concentrations of broilers. Similar hypocholesterolemic effects of probiotic bacterial strains on serum lipids of chickens had been reported in other studies. Jin *et al*. [11] found significant reduction in the serum total cholesterol level of broilers fed 1 g kg^-1^ of a multistrain probiotic comprising 12 *Lactobacillus* strains. Kalavathy *et al*. [10], using the same multistrain probiotic mixture, also found reductions in the serum total cholesterol and triglyceride levels of broilers fed 1 g kg^-1^ probiotic. Mayahi *et al*. [46] fed 0.1% of two commercial probiotics, one consisting of *E*. *faecium* and the other consisting of *Bifidobacterium*, to broilers and found significant reduction in their serum total cholesterol and triglyceride concentrations. Mansoub [47] also reported significant decline in serum total cholesterol and triglycerides of broilers fed 0.5% *L*. *casei*, 1% *L*. *casei*, 0.5% *L*. *acidophilus* or 1% *L*. *acidophilus*.

Supplementation of the three *L*. *salivarius* strains to broilers significantly reduced their serum LDL-cholesterol, but not their HDL-cholesterol levels at 21 and 42 d of age. Similar results of reduction in LDL-cholesterol but not HDL-cholesterol were reported by Kalavathy *et al*. [10] who fed 1 g kg^-1^ of a multistrain probiotic comprising 12 *Lactobacillus* strains to broilers. Panda *et al*. [48] also found a decrease in serum LDL-cholesterol but not HDL-cholesterol in broilers supplemented with *L*. *sporogenes* at 100 or 200 mg kg^-1^ diet. In contrast, Ashayerizadeh *et al*. [49] did not find significant differences in the serum HDL- and LDL-cholesterol concentrations of chickens fed the commercial probiotic, PrimaLac, when compared to control chickens.

Currently, the mechanism(s) responsible for the cholesterol-lowering effect of probiotic is still unclear, but there are several mechanisms, based on reduction of cholesterol synthesis or increase in degradation and excretion of cholesterol [16], that have been proposed. Some probiotic strains with bile salt hydrolase (BSH) activity are able to reduce serum cholesterol through deconjugation of bile salts [17]. Bile acids are secreted into the duodenum in their conjugated forms, however, their deconjugated forms are less soluble and more likely to be excreted from the body, and less likely to be absorbed into the intestine and enterohepatic circulation. Since cholesterol is a precursor for hepatic synthesis of bile acids, the liver needs to synthesise new bile acids from cholesterol in a homeostatic response, resulting in reducing cholesterol. In addition, deconjugated bile acids are known to co-precipitate with cholesterol resulting in more excretion of cholesterol from the body [50]. Gililand *et al*. [51] have also proposed that some lactic acid bacteria are able to assimilate cholesterol into their cells resulting in cholesterol reduction of surrounding environment. Another mechanism for cholesterol-lowering effect of probiotics is their ability to produce intra- and extra-cellular cholesterol dehydrogenase or isomerase for catalyzing the transformation of cholesterol into coprostanol in the intestine [52]. The other enzymatic mechanism for cholesterol reduction activity of probiotic strains is inhibition of HMG-CoA reductase enzyme, an important enzyme for cholesterol synthesis, by probiotic strains [16]. The hypocholesterolemic effect of probiotic strains could also be attributed to their ability to bind cholesterol to their cellular surface [53] and to incorporate cholesterol into their cell membranes toward having a higher cellular resistance against lysis [54].

Feeding 0.5 or 1 g kg^-1^ of a mixture of the three *L*. *salivarius* strains to broiler chickens in the current study did not affect the relative weights of heart, liver, spleen, bursa and pancreas at 21 and 42 d of age. This indicates that the three *L*. *salivarius* strains have no adverse effects on the vital organs and the general health of the chickens. Other researchers [10,26,37,55], using different probiotic strains also did not find any significant differences between the relative organ weights of chickens in the control group and groups receiving probiotics.

In the present study, dietary supplementation of 0.5 or 1 g kg^-1^ of a mixture of the three *L*. *salivarius* strains significantly increased intestinal villus heights of broiler chickens at 42 d of age, and villus height:crypt depth ratios at 21 and 42 d of age. Similar result of increase in intestinal villus heights of probiotic-fed broilers was reported by Awad *et al*. [26]. Peric *et al*. [56] also found that broilers fed a probiotic blend containing *Lactobacillus*, *Bifidobacterium*, *Enterococcus* and *Pediococcus* strains in water, and a probiotic consisting of *E*. *faecium* together with prebiotics fructooligosacharides, cell wall fragments and phycophytic substances in feed, showed significantly higher villi, deeper crypts and higher villus height:crypt depth ratio in their jejunum than broilers of control group at 6 weeks of age, but not at 3 weeks of age.

The small intestine is an important digestive organ involved in nutrient absorption and its development is essential to broiler performance. It has been suggested that probiotic cultures are able to reduce the damage of enterocytes and in turn the demand for enterocytes renewal in the gut [56]. Due to the major role of intestinal microvilli in absorption of nutrients, an increase in the villus height equates to an increase in surface area resulting in more effective absorption of available nutrients [57,58]. Crypts are considered as origin area for production of new epithelial and villus cells. Stem cells at the bottom of crypts divide to form daughter cells, in which one of them is retained as a stem cell, and the other becomes an intestinal epithelial cell. This newly formed cell has to pass the crypt walls and migrate up onto the villus, where it will differentiate further to become a mature, absorptive epithelial cell. Therefore, larger ratio of villus height:crypt depth will result in higher epithelial cell numbers [58] leading to higher absorption of nutrients [57]. Then, higher amounts of absorbed nutrients lead to higher performance and lower FCR [26]. On the contrary, decrease in villus height and increase in crypt depth may lead to poor nutrient absorption, and lower performance [59]. Probiotics may also have effects on the poultry intestine by stimulating it to have more surface for secretion of endogenous digestive enzymes, thus, improving feed digestion and growth performance [56].

Production of harmful enzymes such as β-glucosidase and β-glucuronidase is a safety aspect of probiotic bacteria that needs to be examined. These two enzymes are the major glycosidases in the intestinal tract, which are produced by bacterial strains. These enzymes release toxic metabolites from nontoxic glycosides and prolong the lifetime of toxicants in the body [60]. It has been suggested that bacterial β-glucosidase is responsible for hydrolysis of amygdalin in the gut to produce mandelonitrile, which in turn could be hydrolyzed to produce toxic cyanide [61]. β-Glucuronidase could hydrolyze glucuronides in the gut that can potentially cause the generation of toxic substances [62].

The results of the current study showed a significant reduction in both β-glucosidase and β-glucuronidase activities in the cecal contents of broilers receiving a mixture of *L*. *salivarius* strains (0.5 or 1 g kg^-1^) as compared to control broilers. To date, there is very little information on the effects of probiotics on the activities of β-glucuronidase in chickens. Cole *et al*. [63] reported that young chickens fed yogurt-supplemented diet showed significantly reduced β-glucuronidase activity. Jin *et al*. [60] also reported that feeding *Lactobacillus* cultures to broilers reduced significantly the intestinal and fecal β-glucuronidase, and fecal β-glucosidase activities, but they had no effect on intestinal β-glucosidase activity. Gadelle *et al*. [64] tested 64 *Lactobacillus* strains and found none to be β-glucuronidase-producer. Drasar and Hill [65] reported that almost all strains of *E*. *coli* were able to produce β-glucosidase, but less than 40% of *Lactobacillus* strains had the ability to produce glucosidase. In a recent *in vitro* enzyme assay of the three *L*. *salivarius* strains, we found that none of the strains produced β-glucosidase or β-glucuronidase (unpublished data). Jin *et al*. [60] suggested that the reduction of harmful enzyme activities by *Lactobacillus* strains in the intestine might be due to the partial replacement of the intestinal microflora, especially *E*. *coli* which is a positive producer of the two enzymes, with *Lactobacillus* strains. In the present study, the reduction of β-glucosidase and β-glucuronidase activities in the cecal contents of broilers fed a mixture of three *L*. *salivarius* strains was probably due to the same mode of action by the *Lactobacillus* strains as the results on the enumeration of cecal microbes showed that there was a significant reduction of total aerobes and *E*. *coli* populations and a significant increase of lactobacilli and bifidobacteria populations in the cecal contents.

## Conclusions

The results of this study demonstrated that supplementation of a mixture of three *L*. *salivarius* strains (CI1, CI2 and CI3) at a concentration of 0.5 or 1 g kg^-1^ diet to broiler chickens had similar beneficial effects on them. It improved body weight, body weight gain and FCR, reduced total cholesterol, LDL-cholesterol and triglycerides, increased populations of beneficial bacteria such as lactobacilli and bifidobacteria, decreased harmful bacteria such as *E*. *coli* and total aerobes, reduced harmful cecal bacterial enzymes such as β-glucosidase and β-glucuronidase, and improved intestinal histomorphology of broiler chickens. Their remarkable efficacy on broiler chickens in this preliminary experiment warrants the three *L*. *salivarius* strains to be considered as good potential probiotic for chickens, and their benefits should be further evaluated on a commercial scale.
